# Academic Aspirations of 12th Grade Students in the United States: Place-Based Diminished Returns of Parental Education in Rural Areas

**DOI:** 10.31586/ojer.2025.6040

**Published:** 2025-03-12

**Authors:** Shervin Assari, Gandom Assari, Hossein Zare

**Affiliations:** 1 Department of Psychiatry, Charles R. Drew University of Medicine and Science, Los Angeles, CA, United States; 2 Department of Internal Medicine, Charles R. Drew University of Medicine and Science, Los Angeles, CA, United States; 3 Department of Family Medicine, Charles R. Drew University of Medicine and Science, Los Angeles, CA, United States; 4 Department of Urban Public Health, Charles R. Drew University of Medicine and Science, Los Angeles, CA, United States; 5 Marginalization-Related Diminished Returns (MDRs) Center, Los Angeles, CA, United States; 6 Canyon High School, Anaheim, CA, United States; 7 Department of Health Policy and Management, Johns Hopkins Bloomberg School of Public Health, Baltimore, MD, United States; 8 School of Business, University of Maryland Global Campus (UMGC), Adelphi, MD, United States

**Keywords:** Parental Education, Educational Aspirations, Rural-Urban Disparities, Socioeconomic Status, Minorities’ Diminished Returns, Geographic Differences, Non-Latino White Adolescents, Spatial Inequality, Higher Education, Higher Education, Rural Disadvantage

## Abstract

**Background.:**

The Motivational Theory of Life-Span Development suggests that individual aspirations are shaped by both internal and external resources. Parental education is a key determinant of educational aspirations, yet its effects may vary by geographic location, demonstrating spatial patterns of Minorities’ Diminished Returns (MDRs).

**Objectives.:**

This study examines the association between parental education and aspirations for graduate or professional education among non-Latino White adolescents, with a specific focus on urban-suburban versus rural differences.

**Methods.:**

Using data from the 12th-grade cohort of the Monitoring the Future (MTF) 2024 survey, we conducted multivariate analyses to assess the relationship between parental education and aspirations for graduate or professional education. We further examined whether this association was moderated by geographic location (urban-suburban vs. rural) to identify place-based MDRs.

**Results.:**

Higher parental education was associated with greater aspirations for advanced education; however, this effect was weaker in rural areas compared to urban and suburban settings. These findings highlight that even among non-Latino White adolescents, rural residence diminishes the benefits of socioeconomic resources, providing evidence of place-based MDRs.

**Conclusion.:**

Rural residents face a dual disadvantage—both lower socioeconomic status and weaker returns on those resources—necessitating targeted interventions beyond resource allocation. To address disparities in educational aspirations in rural areas, policymakers should focus on improving equitable access to educational opportunities and ensuring that these resources translate into comparable outcomes across different social and geographic contexts.

## 1. Introduction

Higher education is a critical determinant of economic mobility, career opportunities, and overall well-being [1–4]. Individuals with advanced degrees tend to have better job stability, higher incomes, and improved health outcomes, underscoring the importance of ensuring equitable access to higher education across diverse populations [5–7]. Societies that prioritize educational attainment tend to experience greater social and economic development as well [8–11].

Aspirations for higher education serve as an essential predictor of educational attainment [12–15]. Research suggests that adolescents with high aspirations for graduate or professional education are more likely to pursue and complete advanced degrees [16–18]. These aspirations shape academic effort, resilience, and long-term educational success, making them a crucial focus for understanding disparities in educational outcomes [19,20]. Thus, studying factors that influence educational aspirations can provide insights into mechanisms that shape future socioeconomic success.

Parental education and aspirations for their children [21], as children of highly educated parents tend to have higher educational aspirations [22–25]. Children with lower parental education generally have lower educational aspirations, on average [26,27]. However, the extent to which parental education translates into high aspirations may vary across different social and geographic contexts [28], suggesting that its influence is not uniform for all populations.

Motivation is an important determinant of educational aspirations and achievements. According to the Motivational Theory of Life-Span Development [29–33], individuals are driven by their ability to pursue personal goals, develop competencies, and maintain a sense of control over their environment. Parental education may serve as a key facilitator of motivation, providing adolescents with the confidence and resources needed to set high aspirations for their future education. However, external factors such as geographic location may shape how effectively motivation translates into tangible educational outcomes.

The Minorities’ Diminished Returns (MDRs) framework suggests that resources, such as parental education, generate fewer positive outcomes for some marginalized groups [34]. While high parental education is generally associated with stronger educational aspirations, these benefits may be weaker for certain populations due to structural and contextual barriers [28]. This concept is critical for understanding disparities that persist despite apparent resource availability [35–41].

Most research on MDRs has focused on race [42–48], ethnicity [49–56], immigration status [57–64], and sexual orientation [65,66], highlighting how these factors shape the differential returns of socioeconomic resources. However, limited research has examined how place-based marginalization, particularly rural residence, affects the benefits of parental education. This study addresses this gap by exploring whether rural location functions as a form of social disadvantage that limits the translation of parental education into high educational aspirations.

Understanding place-based MDRs has significant policy implications [46]. If the benefits of parental education are weaker in rural areas, policymakers should go beyond increasing access to education and also focus on ensuring that resources translate equitably into meaningful outcomes [46]. Addressing disparities in educational aspirations may require structural interventions that target both socioeconomic access and the contextual barriers that diminish returns on these investments.

The present study aims to examine the association between parental education and aspirations for graduate or professional education among non-Latino White adolescents, with a focus on urban-suburban versus rural differences. We hypothesize that while higher parental education will be associated with stronger aspirations for advanced education, this effect will be weaker among rural youth, demonstrating place-based MDRs. By identifying these disparities, this study seeks to inform policies that promote equitable educational opportunities across geographic contexts.

## 2. Methods

### 2.1. Data Source and Study Sample

This study utilized data from the 2024 Monitoring the Future (MTF) survey [67–72], a nationally representative survey conducted annually to examine educational aspirations, substance use, and related social determinants among U.S. adolescents. MTF employs a multistage, stratified sampling design to ensure national representation. This study focused exclusively on 12th-grade students, as they are at a pivotal developmental stage where educational aspirations are formed and solidified.

### 2.2. Participants and Sampling

The study included a diverse, nationally representative sample of 12th-grade students from various educational backgrounds, including public, private, and charter schools. The sample also covered a range of geographic locations, including urban, suburban, and rural areas, ensuring that the results capture place-based variations. The final analytic sample consisted of 7,584 non-Latino White adolescents.

### 2.3. Measures

#### Outcome Variable: Aspirations for Graduate or Professional Education:

The primary outcome variable was adolescents’ aspirations to pursue graduate or professional education. Participants responded to a survey item assessing their highest expected level of educational attainment. Responses were measured on an ordinal scale and treated as a continuous variable for analysis.

#### Predictor Variable: Parental Education:

The primary independent variable was parental education level, defined as the highest level of education attained by either the mother or father. Parental education was categorized as follows: (1) Grade school or less, (2) Some high school, (3) High school graduate, (4) Some college, (5) College graduate, and (6) Graduate school. To explore potential spatial MDRs, an interaction term between parental education and rural residence was included.

### 2.4. Covariates

To control for potential confounders, the following demographic and socioeconomic variables were included in the analyses:

- **Age:** Measured in years and included as a continuous variable.
- **Sex:** Coded as male (1) and female (0).
- **Geographic Region:** Classified as Northeast (reference category), Midwest, South, and West.
- **Rural vs. Urban Residence:** Defined according to MTF criteria, distinguishing between rural and urban/suburban settings.

### 2.5. Analytic Approach

To account for the complex sampling design of MTF, survey weights were applied in all analyses to ensure national representativeness. We used linear regression models to assess the association between parental education and aspirations for graduate or professional education, incorporating an interaction term to examine whether rural residence moderates this relationship. All statistical analyses were conducted using Stata 18, employing the svy command to incorporate sampling weights. Statistical significance was set at p < 0.05.

## 3. Results

Table 1 presents the results from Model 1, which examines the relationship between parental education and aspirations for graduate education without interactions. Higher parental education was significantly associated with greater aspirations for advanced education (β = 0.19, p < 0.001), suggesting a strong positive influence of parental education on students’ future educational goals. However, the analysis also revealed disparities by sex and geographic location. Male students reported significantly lower aspirations for graduate education compared to females (β = −0.36, p < 0.001). Additionally, rural residence was associated with lower aspirations for graduate education (β = −0.14, p = 0.005), indicating that students in rural areas have reduced expectations for pursuing higher degrees compared to their urban and suburban peers. Regional differences in aspirations were not statistically significant, as indicated by the non-significant coefficients for the Midwest, South, and West compared to the Northeast (p > 0.05). Additionally, the presence of one or two parents in the household did not have a significant impact on educational aspirations.

Table 2 presents Model 2, which includes the interaction term between parental education and rural residence to assess place-based MDRs. The main effect of parental education remained significant (β = 0.22, p < 0.001), reinforcing its role as a key determinant of educational aspirations. However, the interaction term (Parental Education × Rural Residence) was negative and significant (β = −0.12, p = 0.015), indicating that the benefits of parental education were attenuated for students residing in rural areas. These findings suggest that although higher parental education generally promotes aspirations for graduate or professional education, the magnitude of this effect is weaker in rural settings. This aligns with the MDRs framework, which posits that structural barriers limit the extent to which marginalized groups can leverage socioeconomic resources for upward mobility. In Model 2, the direct effect of rural residence became non-significant (β = 0.39, p = 0.080), suggesting that the observed disparities in educational aspirations among rural students are largely driven by the diminished returns on parental education rather than inherent differences in rural versus urban youth. The patterns of regional effects remained consistent with Model 1, showing no significant variation across U.S. regions.

## 4. Discussion

This study aimed to examine the association between parental education and aspirations for graduate or professional education among non-Latino White adolescents, with a specific focus on geographic differences. The primary hypothesis was that parental education would positively influence educational aspirations, but this effect would be attenuated among rural youth. The study’s findings confirmed this hypothesis, demonstrating that while parental education is a strong predictor of aspirations in general, its impact is less pronounced in rural settings. These results underscore the need for a nuanced understanding of how place-based disparities influence educational trajectories, even within seemingly advantaged groups.

The results indicate that higher parental education is significantly associated with greater aspirations for advanced education among non-Latino White youth. However, this association is notably weaker for those residing in rural areas. This suggests that rural youth do not experience the same level of benefits from their parents’ educational attainment as their urban and suburban counterparts. These findings align with the MDRs framework, which posits that the positive effects of socioeconomic resources are not uniformly distributed across all populations and contexts.

MDRs have traditionally been explored in the context of race, ethnicity, and immigration status. This study extends the MDRs framework by illustrating that geographic location can function as a form of marginalization, limiting the benefits of parental education. Rural adolescents face structural barriers that inhibit the full realization of their socioeconomic potential, reinforcing disparities in educational aspirations and, by extension, future educational and occupational outcomes.

Addressing MDR-related disparities requires distinct and multifaceted policy approaches. It is not sufficient to merely improve access to educational resources; policymakers may also ensure that these resources translate into tangible educational, economic, and health outcomes. This means considering the structural and contextual factors that shape the efficacy of educational investments and tailoring interventions to the specific needs of marginalized communities.

Rural residence often signifies social and economic marginalization due to limited access to quality schools, post-secondary institutions, economic opportunities, and infrastructure [73–75]. These constraints collectively reduce the ability of rural youth to convert parental education into strong aspirations for graduate and professional education. Addressing these challenges requires structural and systemic changes (e.g., enhancing education quality, increasing access to transportation, and high paying jobs) that bridge urban-rural disparities in educational and economic opportunities.

The persistence of geographic disparities in health, education, and employment further compounds the issue. Rural populations face higher rates of poverty, unemployment, and limited access to quality healthcare and social services. These structural inequities create an environment where aspirations for higher education are systematically discouraged, limiting upward mobility [76–78].

Unemployment rates, economic stagnation, and lower educational quality in rural areas present systemic barriers that suppress aspirations for advanced education. Given the scarcity of high-skill jobs in rural areas, young people may see little value in pursuing higher education, as their immediate environment does not offer sufficient opportunities to utilize advanced degrees [79–82].

Although social networks are often seen as a source of support, the network of people in rural areas may not facilitate educational attainment as effectively as networks in urban and suburban communities. In rural settings, limited access to higher education institutions, fewer role models with advanced education, and restricted exposure to diverse career paths can hinder the transmission of educational aspirations and guidance. Many rural families are embedded in networks where higher education is less common or viewed as less necessary, often due to the availability of local jobs that do not require college degrees. Additionally, geographic isolation and underfunded schools reduce opportunities for students to engage with educators, mentors, and extracurricular activities that promote academic achievement. In contrast, urban and suburban students are more likely to be surrounded by peers, educators, and family friends who have pursued higher education, creating a normative expectation for academic success. As a result, rural students may lack both the social capital and institutional resources that are crucial for navigating educational pathways, thereby limiting their ability to pursue higher education.

A potential explanation for the lower aspirations among rural youth is an adaptive response to perceived economic limitations. If adolescents perceive that their employment prospects remain bleak regardless of their educational attainment, they may rationally lower their aspirations to align with available opportunities [83–85]. This highlights the importance of addressing economic disparities alongside educational interventions.

Interventions must be carefully designed to ensure that increases in educational attainment align with available employment opportunities, preventing a surplus of highly educated individuals who are unable to secure appropriate jobs. System-wide changes should be coordinated to avoid barriers in one sector diminishing the benefits of progress in another. Encouraging higher education without simultaneous economic development may lead to underemployment and workforce misalignment, exacerbating frustration and economic stagnation, particularly among rural graduates.

### 4.1. Policy Implications

The findings of this study highlight the need for policy interventions that go beyond simply increasing access to educational resources. While higher parental education is a strong predictor of aspirations for graduate education, the diminished returns experienced by rural students suggest that structural and contextual barriers inhibit the full realization of these benefits. Policymakers should implement geographically targeted initiatives that not only improve educational access but also enhance the effectiveness of these resources in rural areas. This may include investment in rural schools, mentorship programs, and financial incentives to encourage higher education attainment. Additionally, efforts to expand economic opportunities in rural communities may help bridge the gap between educational aspirations and available career pathways, ensuring that higher educational attainment translates into tangible economic benefits for rural youth.

### 4.2. Limitations

Despite its strengths, this study has several limitations that should be acknowledged. First, the reliance on self-reported data on educational aspirations may introduce response bias, as students might overstate or understate their true aspirations due to social desirability or uncertainty about the future. Second, while the study controls for several key demographic and socioeconomic factors, unmeasured variables such as school quality, family support dynamics, and peer influences may also play a role in shaping educational aspirations. Third, the cross-sectional nature of the data limits causal interpretations, as it remains unclear whether lower aspirations among rural students are a direct consequence of diminished returns on parental education or other underlying structural barriers. Future studies utilizing longitudinal data could provide deeper insights into the causal pathways driving these disparities.

### 4.3. Future Research Directions

Future research should further investigate the mechanisms underlying the diminished returns of parental education on educational aspirations among rural youth. Longitudinal studies tracking students from adolescence into adulthood would provide a clearer understanding of how these disparities evolve over time and whether they translate into actual differences in educational attainment and career outcomes. Additionally, research should explore potential moderating factors such as school resources, access to role models, and community-level economic conditions that may buffer or exacerbate the effects of parental education. Comparative studies examining similar trends in other marginalized groups, including racial and ethnic minorities, may also shed light on broader patterns of diminished returns and inform more inclusive policy interventions.

## 5. Conclusion

This study provides evidence that while parental education is a key determinant of adolescents’ aspirations for graduate or professional education, its benefits are not uniformly distributed across geographic locations. Rural students experience diminished returns on parental education, suggesting that structural inequalities limit the extent to which socioeconomic resources translate into higher aspirations. These findings underscore the need for policy interventions that address both access to education and the contextual barriers that influence its effectiveness. Future research should continue to explore place-based disparities in educational outcomes, with the goal of informing more equitable policies that support all students, regardless of geographic location.

## Figures and Tables

**Table 1.** Summary of Survey Linear Regression Model (Model 1)

	OR	SE	95%	CI	p
Age (18 Yrs or Above).	−0.02	0.04	−0.10	0.07	0.660
Sex (Male)	−0.36	0.04	−0.45	−0.28	0.000
Rural Area	−0.14	0.05	−0.24	−0.04	0.005
North East	Ref.				
Mid West	0.01	0.06	−0.10	0.12	0.861
South	0.07	0.06	−0.05	0.19	0.274
West	0.02	0.09	−0.16	0.20	0.814
SES parents Present in the household					
None	Ref.				
One	0.05	0.12	−0.19	0.28	0.702
Two	0.16	0.11	−0.06	0.38	0.158
SES Max Parental Education (1–6)	0.19	0.02	0.15	0.23	0.000
Intercept	1.40	0.16	1.09	1.72	0.000

Note: Outcome: Aspirations for Graduate Education (1–4); SES: Socioeconomic Status

**Table 2.** Summary of Survey Linear Regression Model (Model 2)

	OR	SE	95%	CI	p
Age (18 Yrs or Above).	−0.02	0.04	−0.11	0.06	0.574
Sex (Male)	−0.36	0.04	−0.44	−0.28	0.000
Rural Area	0.39	0.22	−0.05	0.83	0.080
North East	Ref.				
Mid West	0.01	0.06	−0.10	0.12	0.881
South	0.06	0.06	−0.06	0.18	0.320
West	0.01	0.09	−0.17	0.19	0.895
SES parents Present in the household					
None	Ref.				
One	0.04	0.12	−0.20	0.27	0.759
Two	0.15	0.11	−0.07	0.37	0.192
SES Max Parental Education (1–6)	0.22	0.02	0.18	0.27	0.000
SES Max Parental Education (1–6) x Rural	−0.12	0.05	−0.21	−0.02	0.015
Intercept	1.26	0.17	0.93	1.59	0.000

Note: Outcome: Aspirations for Graduate Education (1–4); SES: Socioeconomic Status
